# *PIK3CA* c.3140A>G mutation in a patient with suspected Proteus Syndrome: a case report

**DOI:** 10.1002/ccr3.1546

**Published:** 2018-06-02

**Authors:** Virginia Valentini, Veronica Zelli, Piera Rizzolo, Valentina Silvestri, Maurizio Alimandi, Maria Michela D'Aloia, Sandra Giustini, Stefano Calvieri, Antonio Giovanni Richetta, Giovanni Monteleone, Laura Ottini

**Affiliations:** ^1^ Department of Molecular Medicine Sapienza University of Rome Rome Italy; ^2^ Department of Clinical and Molecular Medicine Sapienza University of Rome Rome Italy; ^3^ Department of Internal Medicine and Medical Specialties Unit of Dermatology Sapienza University of Rome Rome Italy; ^4^ Department of Biomedicine and Prevention University of Rome Tor Vergata Rome Italy

**Keywords:** Fibroadipose hyperplasia, molecular testing, next generation sequencing, overgrowth syndromes, *PIK3CA*

## Abstract

We present a patient with suspected Proteus Syndrome, an overgrowth disorder associated with *AKT1c.49G>A* mutation. NGS analysis detected *PIK3CAc.3140A>G* mutation in the patient's affected tissue allowing for PROS (PIK3CA‐related overgrowth spectrum) diagnosis. The overlapping clinical features in overgrowth disorders highlight the importance of molecular testing for a correct diagnosis.

## Introduction

Overgrowth syndromes represent a group of diseases defined by Tatton‐Brown and Weksberg as a “global or regional excess growth compared with an equivalent body part or the age‐related peer group” 1. They are sporadic, nonhereditary disorders characterized by asymmetric overgrowth of body parts, including skeletal and connective tissues and very frequently associated with plantar hyperplasia. These syndromes are due to the occurrence of postzigous mutations leading to a mosaic distribution of the lesions 2, 3. In general, the severity and variability of these disorders depend on the time of appearance of the somatic mutations during the embryonic development and by the number of cells involved 4.

Genomic analyses, conducted over the years in patients affected by a wide spectrum of focal growth abnormalities, have led to a more defined classification of all the syndromes and have identified in the activation of PI3K/AKT/mTOR pathway the genomic background for their clinical expression 5. All the mosaic overgrowth syndromes present overlapping features, but they can now be considered as distinct disorders classifiable on the bases of the molecular lesions that upregulate the activity of the PI3K/AKT/mTOR pathway 6. They include the Proteus Syndrome (PS), which is typically due to the *AKT1* c.49G>A (p.Glu17Lys) activating mutation 2, the PIK3CA‐related overgrowth spectrum (PROS) and the PTEN hamartoma tumor syndrome (PHTS) 4. The first characterized disorder causing asymmetric tissue overgrowth was the PS 7. PS showed the first compilation of a complete guideline for the evaluation of the diagnosis made up of general and specific criteria (National Institutes of Health in Bethesda, MD, 1998) including three major characteristics: the mosaic distribution of the lesions, their progressive course, and the sporadic occurrence of the disease 8. PROS refers to all patients with somatic mosaic mutation in *PIK3CA* gene, but includes a wide spectrum of disorders with overlapping clinical manifestations, such as Congenital Lipomatous Overgrowth, Vascular Malformations, Epidermal Nevi and Spinal/Skeletal Anomalies and/or Scoliosis (CLOVES) 9, megalencephaly‐capillary malformation (MCAP) and hemimegalencephaly (HMEG) syndromes 10, 11, Type I macrodactyly 12, fibroadipose hyperplasia (FH) 13, and other few clinical disorders 5.

In this study, we describe the molecular genetic analyses carried out in a patient who came to our observation to validate the clinical diagnosis of suspected PS.

## Clinical Report

**Figure 1:**
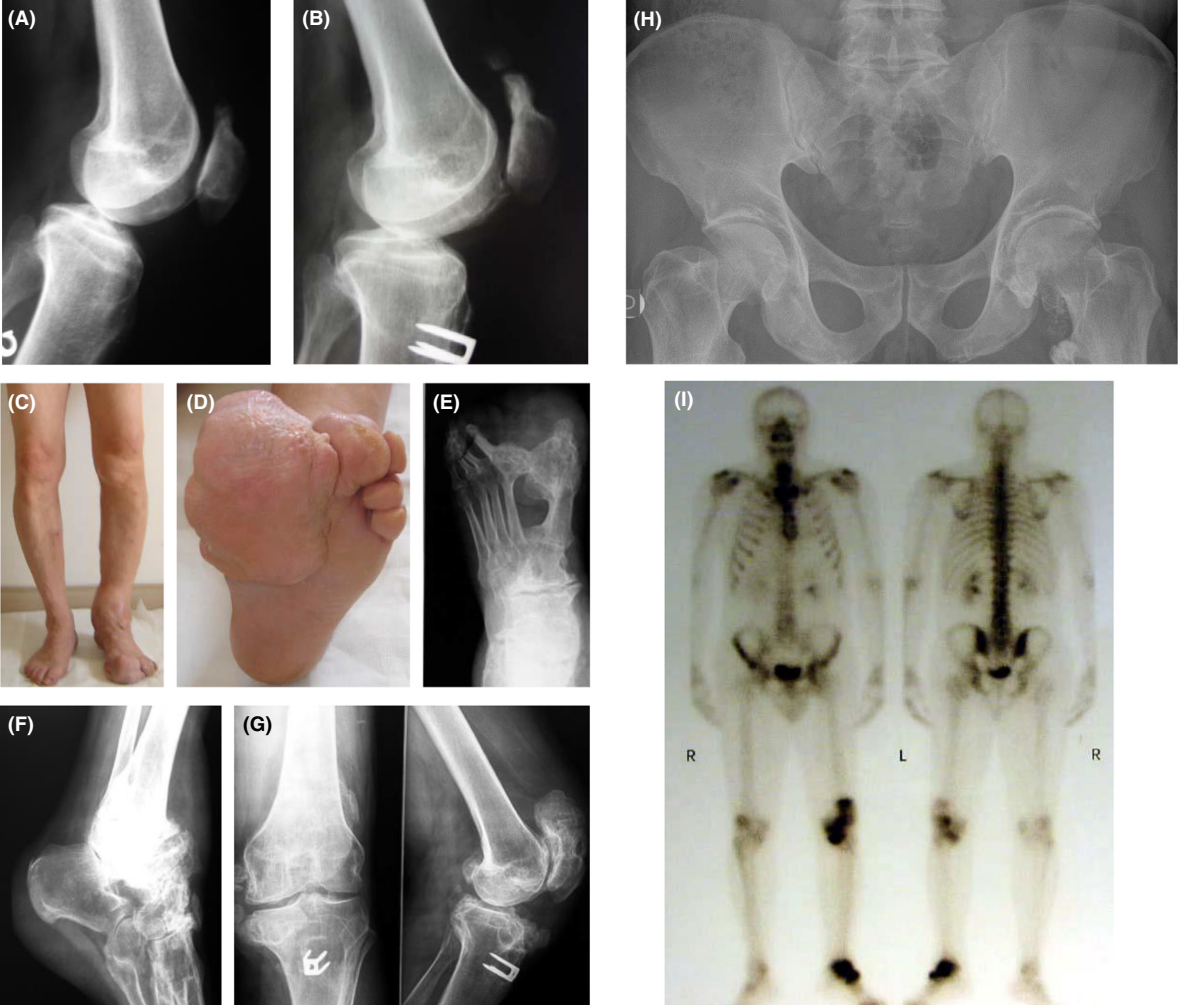
(A–B) X‐ray exam (lateral view) of the knee at the age of 28 years, before surgery (A), and at the age of 30, after the surgical treatment (B). (C–H) Clinical and radiological features at the age of 57: note the disproportion between the two limbs (C) and the hyperplasia on the left sole (D). X‐ray exams show bone productive lesions at the left foot, with partial cutaneous syndactyly between the 1st and the 2nd toes (E), in the ankle (F), hyperostosis at the level of the knee (G), and at the femoral neck and small trochanter (H). (I) Bone scan showing increased uptake in the left ankle and knee.

We report the case of a 57‐year‐old man who came to our attention for an asymmetric segmental overgrowth of the left foot and ankle, characterized by the presence of bone and connective tissue hyperplastic lesions, suggestive for the PS. His medical history began at birth, for the congenital presence of a macrosomia of the left foot and a progressive asymmetric overgrowth of the 2nd toe. At the age of four, the patient was treated with the subamputation of the 2nd toe. At the age 29, he started to suffer from a left patellofemoral pain syndrome, for which he underwent capsuloplasty surgery, with vastus internus reinsertion and patellar tendon transposition with two metal cambers. The X‐ray examination, performed prior surgery, showed incipient osteoproductive lesion in the upper pole of the patella (Fig. 1A). Of note, this lesion appeared greater in volume at the X‐ray examination performed 1 year after surgery (Fig. 1B). At the age of 34, the patient underwent surgery for subtotal amputation of the big toe of the left foot and resection of the calcifications in the dorsal surface of the foot. At physical examination, performed a few years later, the patient showed macrosomia of the left foot and ankle, varus leg deformity and lumps around the knee, and hyperplasia on the left sole (Fig. 1C and D). Tibiotarsic joint had ankylosed, while knee joint showed severe restriction. The X‐ray examination showed bone tissue overgrowth of the left foot and ankle and osteoproductive lesions in the left knee, femoral neck, and small trochanter (Fig. 1E–H). Over the years, foot and knee bone lesions slowly rose in volume. A whole‐body bone scan with technetium‐99 m (Tc‐99 m) showed an increased tracer uptake in the left ankle and knee (Fig. 1I). A magnetic resonance angiography (MRA) of the intracranial vessels and a Doppler examination of the epi‐aortic and large blood vessels excluded the presence of pathological lesions of the arterial and venous vessels.

Despite the absence of characteristics that are common in PS, such as vascular malformations and cerebriform connective tissue lesions, the clinical and radiological signs in our patient suggested a possible diagnosis of PS, according to the general and specific criteria elaborated by the National Institutes of Health (Bethesda, MD) in 1998 8. The patient has no family history of bone productive lesions or overgrowth syndromes and has no signs of neurological or mental abnormalities. He is leading a normal lifestyle and he is father of two healthy children.

## Methods

A peripheral blood sample and two cutaneous biopsies from both affected and unaffected tissue samples of the left foot were collected. Affected tissue refers to the presence of hyperplastic lesions of the connective tissue, whereas unaffected tissue refers to the absence of signs of overgrowth. DNA from blood and fresh frozen tissue samples was extracted using ReliaPrep Blood gDNA Miniprep System (Promega, Madison, WI), and QIAmp DNA Mini kit (Qiagen, Hilden, DE), respectively, according to the manufacturer's instructions. Allele quantification analysis by pyrosequencing using Pyromark Q24 (Qiagen) platform was performed in order to identify the *AKT1* c.49G>A (p.Glu17Lys) mutation. DNA from all patient samples (blood and tissues) as well as DNA from blood of four negative controls was examined. A custom assay (PyroMark Customer Assay, Qiagen), including amplification and sequencing primers designed by Pyromark Assay Design 2.0 Software (Qiagen), was used to evaluate the wild‐type (G) and mutated (A) allele frequency. A standard pyrosequencing sample preparation protocol was applied 14 and results were analyzed by PyroMark Q24 Software (Qiagen), by which the percentage of mutant allele in samples analyzed was obtained. DNA from blood and tissue samples of the patient was also examined by a custom gene panel including the whole coding sequence of *AKT1, PIK3CA,* and *PTEN* genes using Illumina technology. Briefly, paired‐end libraries were prepared using Nextera Rapid Capture Custom Enrichment kit (Illumina, San Diego, CA), pooled and loaded into a MiniSeq System (Illumina) for sequencing and data analysis, including variant calling. Variant annotation and filtering were performed with the Illumina Variant Studio Software. A minimum of 95% of the on‐target regions was covered to a depth of at least 400x. Putative mutations were visualized with the Integrative Genomics Viewer (IGV) tool using hg19 as reference genome and validated by Sanger sequencing using the BigDye Terminator v.2.1 Cycle Sequencing kit (Life Technology, Carlsbad, CA), following the manufacturer's protocol. Primer sequences used for pyrosequencing and Sanger sequencing are available upon request. Variants were named according to Human Genome Variation Society nomenclature (HGVS, http://www.hgvs.org).

After a detailed description of the study, the patient's informed consent was obtained. The study was approved by Local Ethical Committee (Sapienza University of Rome, Prot. 669/17).

## Results

As a first step, allele quantification analysis by pyrosequencing was performed to investigate the presence of the *AKT1* c.49G>A (p.Glu17Lys) activating mutation, specifically associated with PS. Mutational analysis was performed in DNA from blood, affected and unaffected tissue samples of the patient and DNA from blood samples of healthy controls. The *AKT1* c.49G>A (p.Glu17Lys) mutation was not detected in any of the samples analyzed (data not shown).

Therefore, the mutational analysis was extended to the entire coding region of *AKT1, PIK3CA* and *PTEN* genes. DNA from patient's blood, affected and unaffected tissue samples were analyzed by a custom gene panel, comprising these three genes, using NGS technology.

**Figure 2:**
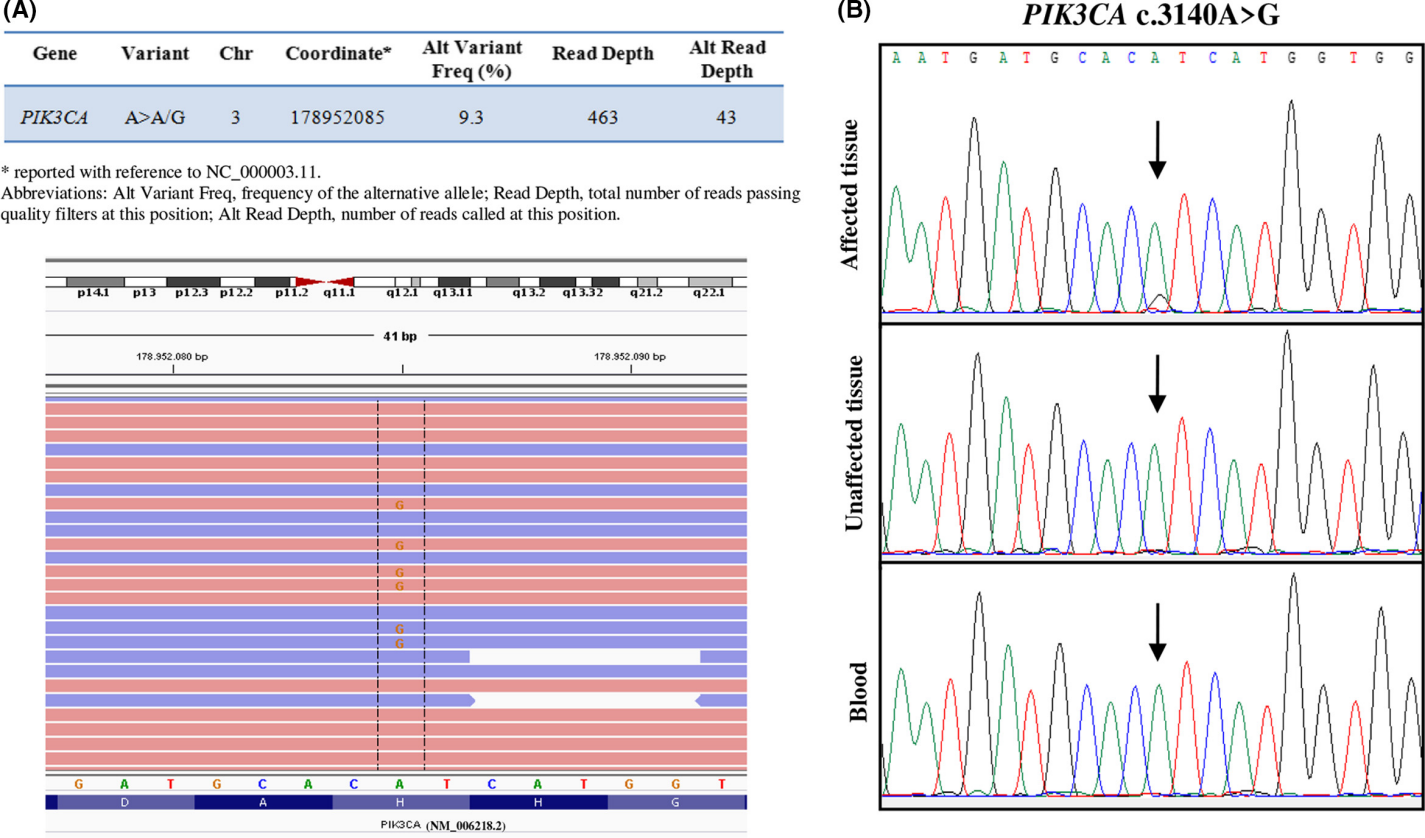
(A) Information about the *PIK3CA* c.3140A>G mutation in affected tissue sample provided by Variant Studio Software (Illumina) and Integrative Genomics Viewer (IGV) visualization; forward and reverse reads are shown in red and blue, respectively. (B) Partial electropherogram of *PIK3CA* confirming the presence of *PIK3CA* c.3140A>G in the affected tissue sample only.

A *PIK3CA* mutation, the c.3140A>G (p.His1047Arg), was identified in the affected tissue sample. In particular, position 3140 was analyzed in a total of 463 reads in both strands and the variant was identified in 43 of these reads, indicating a variant allele frequency of 9.3% (Fig. 2A). The mutation was confirmed by double‐stranded Sanger Sequencing (Fig. 2B). The mutation was not detected in patient's blood and unaffected tissue samples. No mutations were identified in *AKT1*, confirming the absence of *AKT1* c.49G>A (p.Glu17Lys), and *PTEN* genes.

## Discussion

In the present study, we described a patient affected by focal overgrowth of the left foot and ankle, and orthopedic complications with significant clinical morbidities compatible with a diagnosis of PS.

To date, the diagnosis of PS is validated by the presence of the c.49G>A (p.Glu17Lys) mutation in *AKT1* gene 2. The identification of this mutation is necessary because of the wide court of clinical manifestations that are shared in patients affected by focal tissue overgrowth syndromes. For this reason, we first searched for the *AKT1* c.49G>A (p.Glu17Lys) mutation; however, allele quantification analysis, performed using pyrosequencing, excluded the presence of this mutation in our patient, thus leading to the exclusion of the PS diagnosis. Then, we focused on additional genes described to be mutated in other overgrowth syndromes, such as *PIK3CA* and *PTEN* genes. We analyzed the entire coding region of *AKT1, PIK3CA,* and *PTEN* genes by NGS technology using a custom gene panel. This analysis identified a single point mutation, c.3140A>G (p.His1047Arg) in the *PIK3CA* gene, only in the affected tissue sample of the patient, with a frequency of mutant allele of 9.3%.

The *PIK3CA* gene encodes for the 110‐kD catalytic alpha subunit of PIK3, and activating mutations, such as *PIK3CA* c.3140A>G, result in increased downstream catalytic activity, promoting cell survival and proliferation through the increase of AKT and mTOR signaling 5. *PIK3CA* somatic mutations are frequently associated with a variety of solid tumors including breast, colon, lung, ovarian, and gastric cancer 15, 16, 17 and they are also observed in patients with overgrowth syndromes, categorized as PROS 18. Somatic mosaic *PIK3CA* mutations can affect different tissues. The number of cells that carry the mutation can be variable, and the extension and severity of the lesions may range from small areas of overgrowth resulting in isolated disease, such as macrodactyly or megalencephaly, to syndromes defined by tissue overgrowth, vascular malformations and epidermal nevi 4. Thus, PROS includes syndromes with a highly variable clinical presentation, such as CLOVES syndrome, FH, macrodactyly and megalencephaly/hemimegalencephaly syndromes 5.

Collectively, the identification of the *PIK3CA* c.3140A>G (p.His1047Arg) mutation in affected tissue sample and the clinical features of our patient support the diagnosis of FH, syndrome characterized by congenital, progressive and segmental overgrowth of fibrous and adipose tissue and bone 13. Notably, in about 90% of patients affected with PROS that do not present brain overgrowth, five recurrent *PIK3CA* mutations, including *PIK3CA* c.3140A>G (p.His1047Arg), were identified 18. Furthermore, among these five mutations, those affecting codon 1047, specifically p.His1047Arg and p.His1047Leu, are the most frequently observed in FH 19.

Due to the occurrence of overlapping clinical manifestations among many of the overgrowth syndromes, our study highlights the importance of considering and evaluating more than one gene of AKT/PIK3CA/mTOR pathway, in order to identify the genetic component responsible for overgrowth disorder in each patient. In this context, NGS‐based gene panels represent a rapid and cost‐effective technology to analyze multiple genes simultaneously 20. This approach, widely used for mutational analysis in fields such as oncology 21, can also be applied to the molecular diagnosis of genetic disorders such as overgrowth syndromes 22.

The identification of the genetic alteration causing specific overgrowth disorders can have important implications also for treatment strategies. To date, therapeutic approaches for patients affected by overgrowth syndromes are limited to the surgical removal of affected tissue in order to slow down its growth. The identification of the genetic basis of mosaic disorders may be essential to improve patient management through the use of target drugs that may help slow down or even reverse symptoms in patients 23. In this regard, it is noteworthy that several small‐molecule inhibitors of the PI3K/AKT/mTOR pathway, currently used in clinical trials as therapeutic agents for cancer treatment, could also be used for the treatment of segmental overgrowth conditions. This hypothesis is now being tested on the first clinical trial currently ongoing 5.

In conclusion, our results highlight the importance of molecular testing by NGS for an accurate diagnosis and management of patients with overgrowth disorders, as they may be affected by overlapping and confounding clinical characteristics.

## Authorship

VV and VZ: performed experiments, drafted the manuscript, reviewed the manuscript, and agreed to submit the manuscript for publication. PR and VS: interpreted the data, critically revised and approved the manuscript. MA, MMD, SG, SC, and AGR: characterized the patient for clinical features and critically revised and approved the manuscript. GM and LO: involved in the study concept, design, and coordination, interpreted the data, drafted the manuscript, reviewed the manuscript, and agreed to submit the manuscript for publication.

## Conflict of Interest

None declared.
